# Cyanobacterial Toxins as Allelochemicals with Potential Applications as Algaecides, Herbicides and Insecticides

**DOI:** 10.3390/md20080007

**Published:** 2008-05-15

**Authors:** John P. Berry, Miroslav Gantar, Mario H. Perez, Gerald Berry, Fernando G. Noriega

**Affiliations:** 1 Marine Science Program, Department of Chemistry and Biochemistry, Florida International University, 3000 NE 151^st^ Street, North Miami, FL 33181, USA; 2 Department of Biological Sciences, Florida International University, 11200 SW 8^th^ Street, Miami, FL 33199, USA; E-mails: gantarm@fiu.edu, noriegaf@fiu.edu

**Keywords:** Chemical ecology of cyanobacteria, toxins, allelopathy, mosquito larvicide, algaecide

## Abstract

Cyanobacteria (“blue-green algae”) from marine and freshwater habitats are known to produce a diverse array of toxic or otherwise bioactive metabolites. However, the functional role of the vast majority of these compounds, particularly in terms of the physiology and ecology of the cyanobacteria that produce them, remains largely unknown. A limited number of studies have suggested that some of the compounds may have ecological roles as allelochemicals, specifically including compounds that may inhibit competing sympatric macrophytes, algae and microbes. These allelochemicals may also play a role in defense against potential predators and grazers, particularly aquatic invertebrates and their larvae. This review will discuss the existing evidence for the allelochemical roles of cyanobacterial toxins, as well as the potential for development and application of these compounds as algaecides, herbicides and insecticides, and specifically present relevant results from investigations into toxins of cyanobacteria from the Florida Everglades and associated waterways.

## 1. Introduction

In 1959, Gottfried Fraenkel, in his seminal paper, “The Raison D’Être of Secondary Plant Substances,” first proposed that the myriad of secondary compounds from plants – previously considered to be waste products of little importance - might actually serve to determine palatability of these plants particularly with respect to insect herbivores [1]. Several years later, Ehrlich and Raven [2] further proposed that plant secondary metabolites might actually facilitate what they termed the “coevolution” of herbivorous insects and plants. Spawning the field of “chemical ecology,” innumerable studies in the more than four decades that followed have elucidated countless roles of the secondary compounds of plants, insects and other invertebrates, and (to varying extents) other animals and even some microbes, as “allelochemicals” that are involved in the interactions between organisms as it relates to their ecology.

Among the oldest extant organism on Earth, dating back in the fossil record to nearly 3.5 billion years ago, the cyanobacteria (“blue-green algae”) have evolved to produce an impressive array of biologically active compounds. Reviewed elsewhere in this Special Issue on *Marine Toxins* [3], these cyanobacterial metabolites encompass a wide range of chemical classes, particularly including a diversity of nitrogen-rich alkaloids and peptides [4], which have been suggested to both pose threats to human and environmental health worldwide, and equally hold considerable potential for development of pharmaceuticals and other biomedical applications. However, despite this extraordinary evolution of the chemical “repertoire” of cyanobacteria, very little remains known about the functional role of these compounds in the physiology, ecology and natural history of these organisms.

Here we will review some of the relatively limited information that exists with respect to the chemical ecology of cyanobacteria and their biologically active secondary metabolites, focusing particularly on the possible interaction of marine and freshwater cyanobacteria with planktivorous grazers and sympatric algae, as well as the potential for commercial development of these compounds as algaecides, herbicides and insectides. In addition, we will present preliminary data on our research into the possible functional roles of bioactive metabolites from cyanobacteria in the Florida Everglades, particularly in relation to allelopathic interactions with other microalgae, and possible defenses to mosquito larvae, as potential predators of cyanobacteria, in this habitat.

## 2. *Raison D’Être* of Cyanobacterial Toxins?

Cyanobacterial toxins are most well studied, and largely associated, with regard to their effects on human and environmental health. Indeed, toxicity of cyanobacterial metabolites was first reported in the scientific literature by George Francis [5] following death of livestock in South Australia, after consumption of cyanobacteria-contaminated drinking water from Lake Alexandria. Since then, a number of incidents of human and non-human animal poisoning have been described, and several good references on the human health effects of cyanobacterial toxins have been previously published [6–15].

That said, the intoxication of humans and other higher animals is most certainly not the selective force for the evolution of such a diverse repertoire of toxic or otherwise biologically active secondary metabolites from cyanobacteria. A number of possible ecologically and physiologically relevant roles of cyanobacterial secondary metabolites have been previously proposed, and particularly these proposed roles have largely paralleled functions described for higher plants, understandably drawing upon the aforementioned wealth of studies on plant chemical ecology. These roles have primarily included feeding deterrence against potential consumers and competition for resources with sympatric organisms though, to be certain, countless other possible functions could be speculated. Indeed, in this discussion, we may frequently use the term “toxin” to describe these biologically active metabolites which itself implies a negative role of these compounds (though a slightly more refined version of the term is described below). This term is widely accepted and used in the literature, owing to the general association of these compounds with deleterious effects on human and environmental health, particularly including recognized cases of poisoning, as well as to the most commonly asserted ecological role of these compounds in negatively impacting both potential consumers and sympatric competitors.

## 3. Chemical Defenses of Cyanobacteria Against Planktivores

### 3.1 Apparency Theory

When considering the possible role of cyanobacterial toxins in defense against planktivorous grazers, it is perhaps informative to draw parallels to conceptual frameworks set forth in the chemical ecology of terrestrial plants and herbivores. In particular, it is proposed that existing information on the interaction between cyanobacteria and potential grazers seemingly follow (with appropriate modifications, as discussed below) principles related to “apparency theory.”

First put forth by Feeney [16] and Rhoades and Cates [17], apparency theory was one of the first models to provide a conceptual framework for chemical ecology of plants and herbivory. As per the theory, plants such as trees and other perennials that are spatially and/or temporally “apparent” would be unable to escape in space and time, and consequently be more susceptible to, constant herbivory. On the contrary, plants (or even specific parts of plants, e. g. young shoots) which are physically or temporally “unapparent,” due to size, limited distribution and ephemeral nature, would be more likely to escape herbivory, and would have less pressure to invest in large quantities of chemical defenses.

In conjunction with the delineation of apparent and unapparent plants, it was proposed that apparent plants would specifically be expected to produce “quantitative” defenses, whereas unapparent plants would be characterized by “qualitative” chemical defenses. Quantitative chemical defenses would specifically include those that are present in high concentrations, and which would have dose-dependent effects that are generalizable in their action, and largely affect all potential herbivores. In particular, in the case of terrestrial plants, these typically centered on digestion inhibitors, such as tannins and other polyphenolics that non-specifically bind proteins, and therefore have broad activity that is difficult (if not impossible) for herbivores to completely circumvent. Qualitative defenses, generally termed “toxins” in the chemical ecology literature, are those which are typically present at low concentrations in cells (i.e. less resource investment), but that have highly specific modes-of-action, and are effective at relatively much lower doses. As a corollary of this, it is expected that quantitative defenses, given their generalized activity, would be effective deterrents for all potential herbivores, while qualitative toxins, though effective against generalists, would lead to co-evolution of specialist herbivores that are adapted to tolerate or otherwise deal with the specific toxicity. It should finally be noted that apparency theory is generally considered with intrinsically related, underlying principles of resource allocation, such that investment in chemical defenses would be further determined by availability of resources, and ability to allocate necessary resources to synthesis of these metabolites. Details of resource allocation hypotheses, however, will not be discussed to any extent here for simplicity, and due to reasoning that is discussed below.

### 3.2. Apparency Theory and Chemical Ecology of Cyanobacteria

In developing apparency theory as a framework for cyanobacteria and planktivorous grazers, there obviously arise a number of considerable differences from terrestrial plants and herbivores. In particular, unlike true plants, prokaryotic cyanobacteria include representatives of an arguably wider diversity of growth habits ranging from “plant-like macrophytes” to colonial microalgae to spatially large “algal blooms” of unicellular clones, as well as a myriad of symbiotic forms. Accordingly, this rather unique diversity of growth habit would be expected to lead to important distinctions with respect to apparency to potential grazers. Furthermore, unlike terrestrial plants, some aquatic cyanobacteria do exhibit limited ability to physically (albeit slowly) escape grazing, specifically by means of specialized gas vacuoles that allow vertical movement in water-column [18].

In addition, it is proposed that limitations related to resource allocation would also be expected to differ considerably from those of terrestrial plants. Specifically, occurrence of cyanobacteria (except in relatively rare cases) is generally ephemeral, and their proliferation in freshwater habitats is itself generally considered to be dictated by availability of a few limiting nutrients, particularly nitrogen and phosphates [19]. Moreover, in terms of the former, nitrogen-fixation (via specialized heterocysts) by many of the cyanobacteria may further alleviate this limitation. Indeed, as mentioned, nitrogen-rich compounds, and particularly a myriad of non-ribosomal peptides, are characteristic of the cyanobacteria [4]. As such, when cyanobacteria occur in any significant quantity, it is generally assumed that nutrients are not limiting to either algal growth or toxin-production.

Finally, in addition to the notable range of growth habits of freshwater cyanobacteria, there is a concomitant and related diversity in the relative size of potential grazers - ranging from planktivorous fish that may be many orders of magnitude larger than, for example, unicellular cyanobacteria or even colonies, to zooplankton that may be too small and otherwise morphologically unequipped for effective grazing on large colonies, filaments and macrophytic cyanobacteria. To illustrate this, many terrestrial insects are equipped with specialized feeding apparatus (e.g. proboscis, mandibles) for herbivory on much larger plants, whereas most of the grazers of cyanobacteria (e.g. zooplankton) are not so equipped and generally limited to single cells or small colonies of cyanobacteria [20], though cases of grazing on filamentous cyanobacteria have been reported [21–23].

**Figure 1:**
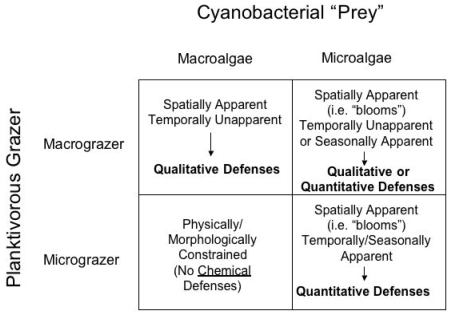
**Proposed Model for Spatial and Temporal Apparancy as it Relates to Chemical Defenses of Cyanobacteria.** Classification of *macrograzers* and *micrograzers*, along with *macroalgae* and *microalgae*, are defined in the text. Cyanobacteria that are spatially or temporally apparent would be expected to employ quantitative defenses, whereas unapparent cyanobacteria would be expected to utilize qualitative defense (“toxins”), as described in the text.

Accommodating these distinctions, Figure 1 proposes a possible scheme for expectations related to apparency and quantitative/qualitative chemical defenses in freshwater cyanobacteria. Though admittedly simplistic, for our purposes cyanobacteria and grazers are classified here into two categories of each, namely “microalgae” and “macroalgae,” and “micrograzers” and “macrograzers.” Specifically, microalgae would include unicellular cyanobacteria, including those that occur in abundance as algal blooms, as well as some smaller assemblages (e.g. colonies) of free-living cyanobacteria; macroalgae, on the other hand, would include those existing as relatively larger assemblages, particularly including various filamentous and “macrophytic” representatives. Micrograzers include those planktivores, such as various taxa of zooplankton grazers, in particular, which are sufficiently large to manipulate cyanobacterial cells, but may be physically or morphologically unable to exploit larger prey, and would also typically be characterized by short-lived, seasonal existence. Macrograzers, such as planktivorous fish, on the other hand, would be capable of consuming both quantities of single-celled algae and potentially larger prey, including macrophytic forms, and would additionally have considerably longer life histories, presumably spanning years.

It should be noted that this simplified classification system, and proposed model of apparency as it relates to chemical defenses, is admittedly not a comprehensive model to explain all interactions between grazers and cyanobacteria in marine and freshwater habitats. The classification system proposed her does not, for example, incorporate mesograzers (e.g. amphipods) that are important planktivorous grazers in marine and freshwater habitats, and can share characteristics of both micro- and macrograzers. Likewise, other aspects of cyanobacterial grazing, such as role of nutritional parameters, are also not discussed here. Indeed, it has been argued that cyanobacteria are relatively limited in nutritional value, specifically with respect to fatty acids and certain sterols [24–26]. Instead, the proposed model is intended solely as a preliminary framework for reviewing the currently existing (and relatively limited) information regarding the suggested, though not yet definitive, role of cyanobacterial chemical defenses with respect to potential micro- and macrograzer pressures.

According to the proposed model (Figure 1), several predictions can be made regarding the expected chemical defenses of cyanobacterial forms with respect to micro- and macrograzers. An immediate assertion of the proposed models, and more accurately a product of the proposed classification of micrograzers as being unable to manipulate larger cyanobacterial forms (e.g. larger colonies, filaments, macrophytes), is that chemical defenses by macroalgae against micrograzers would be unnecessary. As mentioned previously, unlike terrestrial herbivores (e.g. insects) with adaptive features for feeding on plants, there are not (to the authors’ knowledge) similar adaptations in freshwater micrograzers. That said, there are, indeed, limited examples of certain zooplankton consuming filamentous cyanobacteria, and these exceptions would be expected to follow patterns similar to the relationship of micrograzers with spatially and temporally apparent microalgae; examples of these are discussed below (see *Micrograzer-Microalgae Interactions,* below).

Compared with the extensive literature on the role of chemical defenses in plant herbivory, there is a relative paucity of such studies on cyanobacteria in support of the proposed model. As discussed above, it is not an assertion that the examples, given here in support of this proposed conceptual framework, are at all comprehensive. Rather, this conceptual framework is given to underscore the emerging evidence that does exist on possible ecological roles of cyanobacterial secondary metabolites, and to demonstrate the seeming consistency of this data with the proposed model of apparency as it relates chemical defense.

### 3.3. Macrograzer-Macroalgae Interactions: Qualitative Toxins of Lyngbya majuscula

An overriding assumption regarding cyanobacteria is that they are, regardless of growth habit, generally ephemeral (depending on nutrients and other growth conditions) in their occurrence, and therefore may be considered temporally unapparent, specifically to macrograzers (e.g. fish) whose life histories may span several years. In particular, cyanobacteria in freshwater and marine systems are generally restricted by nutrient availability and, moreover, seasonal temperature [19]; for example, blooms of cyanobacteria in temperate zones most typically occur during limited, warm summer months. Given this general lack of apparency in time, both microalgae and macroalgae would be expected to primarily produce qualitative defenses against potential macrograzers.

In terms of macrograzer-macroalgae interaction, perhaps the best example of a documented role of cyanobacterial toxin from physically apparent cyanobacteria in aquatic environments is the marine species, *Lyngbya majuscula. Lyngbya* as a genus produces a particularly diverse array (well over one hundred characterized so far!) of biologically active metabolites [4,15, 27]. Though not truly multicellular, *L. majuscula*, sharing the filamentous growth form of the genus, forms physically apparent “macrophytic” populations in reef and other benthic habitats. The occurrence of *L. majuscula* is, however, largely ephemeral [28–29] such that these macrophytic “blooms” can be largely characterized as unapparent in time, and accordingly would be expected to produce qualitative defenses.

**Figure 2:**
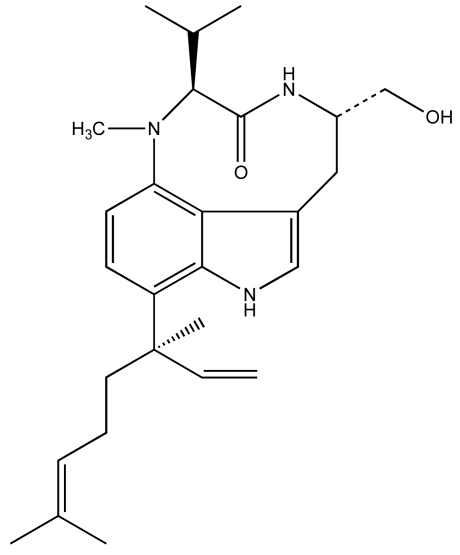
Chemical structure of lyngbyatoxin A.

Considerable work, particularly by Valerie Paul and colleagues [29–38] have specifically elucidated a rather intriguing picture of the *L. majuscula*-derived toxin, lyngbyatoxin A (LTA; Fig. 2), and its role in the ecology of this species. LTA, a specific activator of protein kinase C, is most commonly associated with contact dermatitis resulting from human exposures to *L. majuscula*, however, has been shown to be toxic to fish and an array of potential grazer species [39]. In keeping with its role as a qualitative defense, the potent and specific toxin was recovered (in initial isolations) at rather low yields on the order of 0.02% dry weight [39], suggesting a low cellular concentration. Subsequent studies [35, 37] have shown that crude extracts, containing LTA, in artificial diets significantly deterred feeding by generalist grazers including rabbitfish (*Siganus fuscescens*), as well as several invertebrates including amphipods (e.g. *Parhyale hawaiensis, Cymadusa imbroglio*), urchins (*Echinometra mathaei*) and crabs (*Menaethius monoceros*). Likewise, generalist grazers, such as rabbitfish and green turtles (*Chelonia mydas*), generally avoid grazing on *L. majuscula* containing LTA [29, 32–33, 36, 40–41]. These results are again consistent with avoidance of target-specific toxins (i.e. qualitative defenses) by potential grazers.

In keeping with the qualitative nature of LTA, specialist grazers, most notably including the sea hare, *Stylocheilus striatus*, preferentially graze and grow well on diets of LTA-containing *L. majuscula*, and are actually stimulated to feed by crude LTA extracts, even without prior exposure to the toxin [36]. Furthermore, *S. striatus* is actually found to sequester LTA, particularly in digestive gland, as well as ink, fecal matter and body tissue [42–43]. Though the possible role of this sequestered toxin in defense of the sea hare is unclear [44–45], it is noteworthy that there have, indeed, been several similar cases of putative cyanobacterial (and other algal) origins for toxins isolated from sea hares [46–47]. Again, the apparent adaptation of specialist grazers capable of not only tolerating, but also preferentially feeding on, this otherwise toxic cyanobacterium is consistent with expectations of apparency theory.

These studies collectively suggest a role of LTA generally in the chemical ecology of *Lyngbya*, and specifically as a qualitative feeding deterrent, providing a competitive advantage with respect to grazing pressures in these benthic habitats. It should, however, be further noted that, in addition to LTA, *L. majuscula* is known to produce a suite of chemically related non-ribosomal peptides, including ypaoamide, malyngolide, the malyngamides and pitipeptolide A, and similar contributions to feeding deterrence, and sequestration of these toxins, has been documented for these [29, 32–33, 38, 40]. This may be particularly relevant, given the recognized intraspecific variability in the production of toxins by cyanobacteria, as it might provide a possible underlying mechanism for adaptation between the toxin-producing algae and specialist grazers at the population level.

### 3.4. Micrograzer-Microalgae Interactions: Quantitative Defenses of Microcystis aerugunosa Against Daphnia

Cyanobacterial blooms are generally ephemeral, and as such might be largely considered as unapparent in time, particularly with respect to macrograzers (though exceptions to this are discussed further in the next section). Unlike macrograzers, however, zooplankton micrograzers in freshwater systems, particularly comprised of various adult invertebrate microfauna (e.g. microcrustaceans, rotifers) and invertebrate larvae, are themselves largely characterized by short generation times and seasonal fluctuations, and, moreover, these annual cycles of their occurrence widely overlap with blooms of microalgae specifically during summer months in temperate habitats. Thus, it is proposed that cyanobacteria would be apparent in space and time to micrograzers that can (and do) co-occur with blooms. Accordingly, it would be expected that microalgae would produce quantitative defenses against spatially and temporally co-occurring micrograzers.

Perhaps the best emerging example of chemical defenses in micrograzer-microalgae interactions is the apparent role of the cyanobacterial toxins against grazing by the freshwater microcrustaceans, particularly including the cladoceran genus, *Daphnia*. Cyanobacteria represent a significant component of the daphnid diet [48] despite their reportedly low nutritional value [24–26]. It has been widely demonstrated, however, that ingestion of various toxin-producing cyanobacteria can actually lead to mortality and decreased growth rate of these daphnids [48–59]. Though further evidence is certainly required to conclusively demonstrate the role of these metabolites in the chemical defense against these micrograzers, a considerable amount of research has investigated the role cyanobacterial toxins in this system.

**Figure 3:**
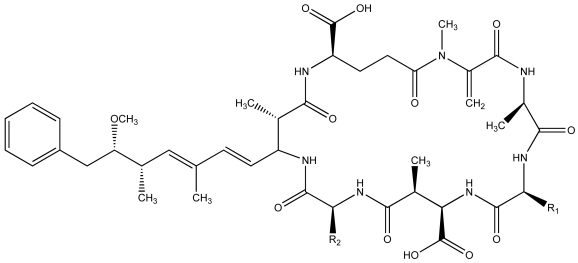
**Generalized Structure of the Microcystins.** The *X*- and *Z*-positions are occupied by various amino acids; these positions are occupied by lysine (L; R_1_ = CH_2_CH(CH_3_)_2_) and arginine (R; R_2_ = CH_2_CH_2_CH_2_NHC(NH)NH_2_) in microcystin-LR, typically the most common of the more than 70 variants.

Initial studies [49–52] focused, in particular, on the widespread *Microcystis aeruginosa* that is known to produce the well-described microcystins (Figure 3). These non-ribosomal peptides are inhibitors of serine/threonine protein phosphatases 1 and 2a (PP1/2a), and most frequently are associated with hepatotoxicity in mammals and other vertebrates [60–61]. In fact, studies with genetically engineered mutants [62], and other strains of *M. aeruginosa,* that do not produce microcystins or lack particular variants (of the more than seventy described), have clearly shown that microcystins (including specific variants) contribute to the lethal effects on daphnids and related zooplankton [54–56, 63].

These lethal effects of microcystins toward micrograzers, in part, though may be considered quantitative in nature. Though microcystins inhibit specific subclasses of serine/threonine phosphatases, the type 1 and 2A subclasses include countless representatives with a broad range of targeted protein substrates in nearly every type of organism [64]. Accordingly, it is asserted that the microcystins may not be classified as target-specific toxins *per se*, but rather quantitative inhibitors of a wide array of potential targets. Also consistent with microcystins as quantitative defenses, they can and do exist at rather high intracellular concentrations in *Microcystis*, frequently on the order of milligrams per gram of dry weight [65]. Indeed, numerous studies have indicated that lethal effects associated with exposure of *Daphnia* to both dissolved microcystins [66] and *M. aeruginosa* cells [54–56, 63] are quantitative.

Moreover, subsequent evidence suggests that toxicity to microcrustaceans is not correlated with PP1/2a inhibition or microcystin concentration [67]. Specifically, in addition to lethal effects, *Microcystis* presented in the diet has been shown to inhibit feeding by *Daphnia,* leading to a quantitative decrease in the growth of the micrograzer population [50, 54, 56, 68], and several studies have, likewise, shown that metabolites from *Microcystis* and other cyanbacteria act as digestion inhibitors in daphnids [69–71] and other microcrustaceans [72]. Consequently, it has become clear from these studies that while microcystins may be involved as qualitative toxins or arguably quantitative inhibitors when present, quantitative inhibition of feeding and digestion by dietary *Microcystis* is not correlated with microcystin concentrations [54–56, 63, 68]. Instead, recent studies have pointed to other digestion and feeding inhibitors from *Microcystis.* In particular, various peptides, including the cyanopeptolins, micropeptins and microviridins, that appear to inhibit trypsin-like proteases, have been largely associated with digestion inhibition [69–74].

With respect to the proposed quantitative nature of the chemical defenses of *Microcystis* against *Daphnia*, it is important to note an emerging body of evidence that suggests adaptations of *Daphnia* and other micrograzers for tolerance to microcystins [75–80]. Adaptation of *Daphnia* to potentially toxic cyanobacteria was perhaps first suggested by Hairston *et al.* [75–76] who demonstrated that *D. galeata* hatched from dormant eggs, specifically collected from sediments of Lake Constance, showed patterns of tolerance to toxic effects of *Microcystis* (as measured by decreased effects on growth rate) that correlated with periods of heavy eutrophication. Specifically, it was found that *Daphnia* from eggs obtained from sediments, representative of periods immediately following intense eutrophication, were less susceptible to diet containing a toxic *Microcystis aeruginosa* isolate than those collected from periods during or prior to this episode [75]. Following this, Gustafsson and Hansson [77–78] experimentally demonstrated that prior exposure of *D. magna* to diets containing toxic *Microcystis* increased survivorship, growth and reproduction in subsequently exposed generations, suggesting an acquired genotypic tolerance to *Microcystis* toxins. Similarly, Sarnelle and Wilson [79] showed that populations of *Daphnia* isolated from highly eutrophic lakes, likewise, had a higher tolerance to diets of toxic *Microcystis* than those from less eutrophic lakes. Specifically, it has been suggested subsequently [80] that *Daphnia* may up-regulate tolerance levels to toxic *Microcystis* in response to the presence of cellular cues from the cyanobacterium, other than microcystin, in keeping with the possible role of non-microcystin metabolites.

Though these data do strongly support adaptions for tolerance to *Microcystis* toxins, and might be taken consequently to argue against the quantitative nature of these compounds, it is equally important to note that currently available data on the mechanism of this tolerance to *Microcystis* toxins suggest likely “detoxification” of these compounds [81–83] rather than resistance via adaptation of the toxin target (e.g. protein phosphatases, proteases) as would be characteristic of a qualitative toxin. Indeed, similar scenarios are evidenced in chemical ecology of herbivores and terrestrial plants. For example, good deal of evidence supports the ability of terrestrial herbivores to detoxify even the tannins (as “classic” examples of quantitative defenses of terrestrial plants), particularly through salivary proteins that preempt the binding of these tannins to digestive enzymes [84–85]. However, such an adaptation specifically limits the bioavailability of the chemical defenses in active form, but does not involve adaptation of the toxin’s target (i.e. resistance). Accordingly, it is argued that though *Daphnia* may, likewise, adapt to the presence of quantitative cyanobacterial defenses, this adaptation is likely not related to co-evolution in terms of the toxin and target, as described for truly qualitative toxins, but rather based on mechanism(s) which remove or detoxify these compounds. Specifically, it has been suggested by Pflugmacher [81], and further supported by others ([82–83], that tolerance to microcystin by cladocerans (and various other plant and animal species) occurs via detoxification of microcystins by conjugation, and subsequent elimination, by glutathione, whereas no adaptation of protein phosphatases, as the targets of the microcystins, has been reported. Similar studies on mechanisms of tolerance with respect to apparent feeding and digestion inhibitors (i.e. other than microcystin) from *Microcystis* are, however, currently lacking.

### 3.5. Macrograzer-Microalgae Interactions: Seasonal Apparency and Microcystins as Quantitative Defense Against Freshwater Fish

Though, generally speaking, it is asserted that microalgae are temporally unapparent to macrograzers, this assertion may require some modification in the case of freshwater cyanobacteria. Although blooms of cyanobacteria are not generally persistent year-round in freshwater systems, their seasonal occurrence does make them predictable, and therefore arguably apparent (both spatially and temporally) to potential macrograzers in these habitats. Accordingly, it would be expected that such microalgae might produce quantitative defenses against macrograzers as well. In support of this, several studies in freshwater systems have reported fish kills associated with cyanobacterial blooms, and particularly microcystin-producing *Microcystis* [86–88]. Specifically, studies [86–91] indicate that the toxic effects of microcystins are largely dependent on the amount of toxin ingested, consistent with the proposed quantitative and relatively non-specific inhibition of ubiquitous Ser/Thr protein phosphatases (as discussed above) by microcystins. Though microcystins primarily affect hepatocytes that accumulate the toxin, these effects are themselves not particularly specific in nature, and Fisher and Dietrich [88], for example, documented an array of quantitative, pathological effects on the hepatocytes and other tissues of the cyprinid, carp (*Cyprinus carpio*), specifically at relatively high doses (typically > 1mg/kg). Li *et al.* [90] fed carp (*Cyprinus carpio*) samples of *Microcystis* bloom, containing 50 μg/kg microcystin, and observed a significant decrease in growth of the exposed (vs. control) fish without acute toxicity (i.e. behavioral effects, mortality), suggesting a decidedly quantitative role of the toxin. Likewise, exposure of embryos and larvae zebrafish [91] to dissolved microcystins specifically inhibited growth rates further suggesting a quantitative effect of the microcystins on potential macrograzers.

It is, however, worth noting that this example of freshwater macrograzer-micrograzer interaction sharply contrasts with cases of marine microalgae that are not as seasonably predictable (and, thus, apparent in time). Most notably the “Red Tide” organism, *Karenia brevis*, and its associated toxins, the brevetoxins, have clearly qualititative effects (i.e. rapid mortality and intoxication) on potential planktivorous fish (and a variety of other animals), exerting a potently neurotoxic mode of action, specifically activating certain voltage-gated sodium channels, and resulting in massive fish kills with even relatively low concentrations of the alga and toxin [92].

## 4. Allelopathic Compounds from Cyanobacteria

In addition to the reputed role of cyanobacterial secondary metabolites as chemical defenses against potential planktivores and other grazers, emerging evidence also suggests a role of these compounds in allelopathy. Allelopathy, again perhaps best described in terrestrial plant systems, involves the use of biologically active metabolites by one species to inhibit the growth of sympatric species that might potentially compete for resources. In the case of freshwater cyanobacteria, this would most likely include other photoautrophs, and particularly algae (including cyanobacteria) and even unrelated microbes, as well as possibly aquatic plants, which might compete for nutrients and light that would limit growth of the cyanobacterial populations.

Keating [93] demonstrated quite elegantly that extracellular components from cultures of “dominant” cyanobacteria isolated during succession of a single freshwater pond specifically showed inhibitory effects on predecessor strains, but not successor strains, from this system, supporting a clear role of extracellular compounds in the succession of cyanobacterial populations. Subsequent studies have continued to support the role that these allelochemicals in controlling annual variability in phytoplankton communities [94]. Vardi *et al.* [94], for example, has presented compelling evidence to suggest reciprocal non-nutritional control of population growth between microcystin-producing *Microcystis* sp. and the dinoflagellate, *Peridinium gatunense* in the mesotrophic Sea of Galilee based on apparent allelopathic compounds, including microcystins and unknown components of the *P. gatunense* culture medium.

**Figure 4:**
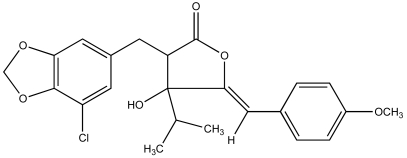
Structure of Cyanobacterin, an Inhibitor of Photosystem II and Proposed Allelopathic Agent Produced by a Freshwater Species of *Scytonema* (Mason *et al.*, 1982).

Consequently, a growing number of studies have identified metabolites from cyanobacteria that act as algaecides [95–97]. An excellent review of these studies has been previously published by Smith and Thanh Doan [98]. Mason *et al.* [95] reported the identification and characterization of a chlorinatedγ–lactone, named cyanobacterin (Figure 4), from a freshwater species of *Scytonema* that specifically inhibited a range of algae, including cyanobacteria and green algae, at micromolar concentrations, but had little effect on non-photosynthetic microbes. It was later found that cyanobacterin specifically inhibits photosystem II [99]. Several years later, Vepritskii *et al.* [96] and Gromov *et al.* [97] reported the identification of compounds from strains of *Nostoc linckia* that specifically inhibited photosynthetic algae. Named cyanobacterin LU-1 and LU-2, these compounds share no significant structural similarity to the previously characterized cyanobacterins. Both compounds, however, inhibited electron transport in photosystem II, and LU-1 was found to be inhibitory to cyanobacteria and other algae, but not non-photosynthetic microbes, whereas LU-2 inhibited cyanobacteria only.

Flores and Wolk [100] and Shlegel *et al.* [101] independently screened sixty-five and approximately two hundred isolates of cyanbacteria, respectively, for algaecidal activity. Interestingly, it was found in these studies that anti-algal activity was largely restricted to several genera, namely *Fischerella, Nostoc, Anabaena, Calothrix and Scytonema,* primarily in Sections IV and V of the standard classification system of Rippka *et al.* [102] that contain nitrogen-fixing, heterocystous filamentous cyanobacteria [100–101]. *Fischerella* produces the fischerellins A and B, as well as a series of indole alkaloids, particularly the hapalindoles, which both inhibit photosystem II [103–104; discussed further below]. The latter have also been shown to inhibit RNA synthesis [105] that has been proposed as alternative mechanism for allelopathy [106]. Accordingly, the pentacyclic calothrixins, isolated from a *Calothrix* species [107], have also been shown to inhibit RNA polymerase, as well as DNA synthesis, and proposed to be involved in allelopathic interactions [106].

In addition to cyanobacterins LU-1 and LU-2, the widespread genus, *Nostoc,* produces several other metabolites that have been associated with algaecidal activity. Nostocyclamide [108] is a cyclic peptide that appears to act as uncoupler of electron transport in photosynthesis [Jüttner 1997 cf. 98]. Identification of the structurally similar nostocyclamide M that also inhibits cyanobacteria [109] suggests these molecules may belong to a family of potentially allelopathic compounds. Hirata *et al.* [110] isolated and characterized a heterocyclic pigment, nostocine A, from an isolate of *N. spongiaforme* that was later found [111] to inhibit green algae, and, to a lesser extent, cyanobacteria. Based on the identification of inhibitory activity against cyanobacteria by Flores and Wolk [100], Becher *et al.* [112] identified the carboline alkaloid, nostocarboline, which was subsequently found to inhibit various cyanobacteria, including the toxic *M. aeruginosa*, specifically by apparent effects on photosynthesis. Moreover, the inhibitory concentration against the producing strain*, Nostoc* 78-12A, was considerably higher than for other cyanobacteria tested, supporting the role of the compound in allelopathy.

In addition, several studies have investigated the possible allelopathic role of microcystins [113–121]. Microcystins have been shown to inhibit cyanobacteria, including *Nostoc*, *Synechococcus* and *Anabaena* species [117], and the green alga, *Chlamydomonas* [116]. Sedmark and Kosi [113] showed that inhibition of phytoplankton by microcystin-RR was light dependent, and subsequently Hu *et al.* [121] recently showed that the compound specifically affected pigment production and photosystem II in cyanobacteria. Interestingly, the presence of the green alga, *Spirogyra,* as well as extracts of the alga, were found to stimulate the production of microcystins by *Oscillatoria agardhii* [122] suggesting a possible production of the compound in response to potential competitors.

A number of the studies on allelopathy related to microcystins have actually focused on inhibition of aquatic plants rather than microalgae [114–115, 118–119, 121]. Pflugmacher [118], for example, demonstrated that microcystin-LR inhibits the growth of several aquatic macrophytes, including species of *Ceratophyllum, Myriophyllum*, *Cladophora*, *Elodea* and even *Phragmites*, specifically affecting pigmentation and photosynthetic oxygen production. Jang *et al.* [121] showed that microcystin-producing *M. aeruginosa* negatively impacted the growth of *Lemna japonica*, and furthermore that the apparent allelopathy was reciprocal, such that exposure to the plant decreased the biomass of the cyanobacteria in culture, and concomitantly increased the production in-turn of the microcystins by these cultures. Casanova *et al.* [114], however, showed that though the presence of high densities of microcystis cells inhibited germination and establishment of representative aquatic plants, the addition of pure microcystin alone had no effect, suggesting other possible factors. In fact, in addition to the microcystins, several other metabolites from *Microcystis* have also been shown to have inhibitory activity against photoautrophs, specifically including kasumigamide [123], microcin SF608 [124] and fatty acids [125–126].

Indeed, other cases of possible allelopathy of cyanobacteria with respect to aquatic plants have also been documented. For example, in addition to inhibiting other microalgae (discussed above), nostocine A was found to inhibit root elongation by barnyard grass [110] suggesting a possible ability to limit colonization of plants that might compete for micronutrients. This is particularly interesting as nostocine A was first isolated from rice paddys [110]. Likewise, fischerellin A that, as discussed above, inhibits photosystem II in algae [103], has a similar activity against the *Lemna minor* with a concomitant inhibition of growth [127]. Entzeroth *et al.* [128] reported that a fatty acid, 2,5-dimethyldodecanoic acid, from the marine cyanobacteria, *Lyngbya majuscula*, inhibited the growth of *L. minor*. It is also worth noting that, though not our focus here, a similar number of reports have shown that metabolites from various higher plants also inhibit the growth of cyanobacteria [129–132].

Though a considerable number of studies have demonstrated *in vitro* activity of cyanobacterial toxins that would suggest potential roles in allelopathy, ecological data to support this role is lacking. In fact, Babica *et al.* [133] has argued that environmental concentrations of these compounds may not be high enough to support the proposed role in allelopathy. Citing microcystins as potential allelochemicals, it was noted that typical environmental concentrations of the toxin are below 10μg/L, but that most inhibition of photoautotrophs by microcystins occurred at concentrations greater than 100 μg/L. However, as pointed out by Hu *et al.* [120], concentrations of microcystin can vary considerably and, in some cases, can exceed 100 μg/L. Moreover, it is noteworthy that presence of both green algae and aquatic plants, including extracts from each, were shown to increase the concentration of intracellular and extracellular microcystin in exposed cyanobacteria [121–122]. Furthermore, though measured concentrations in water samples may not typically exceed 10 μg/mL, there may be localized concentration of the toxin within microenvironments, particularly including those relevant to interactions between microalgae [120]. Though a good deal is known about the bioavailability of microcystins in freshwater systems, little is known about environmental concentrations of most other putative allelochemicals. Generally speaking, therefore, considerably more ecological research is needed to clarify the role of cyanobacterial toxins in allelopathy, as well as other proposed roles.

## 5. Potential Commercial Development of Insectides, Algaecides and Herbicides from Cyanobacteria

Regardless of the arguments for ecological roles of cyanobacterial toxins, it is clear that a wealth of biologically active metabolites exist which may have potential for application to controlling or deterring growth of other organisms. Several studies have investigated, and continue to investigate, the potential biomedical applications of these compounds ranging from antibiotics against microbial infection to treatments for inhibition of cancer cells [e.g. 134]. Likewise, potential commercial development of cyanobacterial compounds for non-biomedical applications, particularly including herbicides, algaecides and insecticides poses a potentially important opportunity to utilize the biological activity of these compounds. In an evaluation of various marine natural products, Peng *et al.* [135] points-out that, in 1997, for example, approximately $6.8 billion and $3.6 billion were spent in the U. S. on insecticides and herbicides, respectively, underscoring the commercial potential of this strategy. Indeed, given the recognized persistence and toxicity of many currently used pesticides, there is considerable interest in identifying effective, “natural” alternatives for such purposes of environmental control (e.g. insecticides, herbicides, algaecides). To date, however, there has been no reported assessment of any such potential application, and discussion of such is, therefore, generally limited to our understanding of *in vitro* activity and limited ecological information. Accordingly, evaluations of both the “pros and cons” of this approach generally, as well as the specific considerations for potential development and application of any candidate compound, are needed. Indeed, investigation of the ecological roles of these compounds will surely be crucial to the possible application of these compounds for such purposes.

To begin with, our understanding of both the advantages (“pros”) and the disadvantages (“cons”) of this proposed approach, in general, are directly related to our understanding of the ecology of these compounds. In terms of advantages of this approach for identifying such compounds, the ecological role of these secondary metabolites in allelopathy and antifeedant activity would both provide a logical “lead” to the desired activities, and an ecological context in which to evaluate environmental implications of these compounds. In the simplest way, the possible ecological roles of cyanobacterial metabolites, as discussed, make them prime candidates for screening and subsequent evaluation studies, particularly compared to random screening of, for example, synthetic libraries of compounds. Furthermore, the “natural laboratory” of these aquatic habitats, where the role of these metabolites as allelochemicals is “played-out,” provides a unique opportunity for insight as to the ecological implications of the proposed application of these compounds, particularly including possible repercussions for ecosystem health (e.g. species displacement). Indeed, consideration of any such agent (whether naturally-occurring or not) that might be used for purposes environmental control (e.g. insecticides, anti-fouling agents, antialgal compounds) would certainly be evaluated equally in these terms. In the case of synthetic compounds, such candidates would lack any pre-existing understanding of such ecological implications. On the other hand, ecological investigation of the role of naturally occurring compounds would provide some degree of *a priori* knowledge as to the possible impacts of these compounds in the relevant aquatic habitats. Additionally, the potential development and use of such compounds from cyanobacteria would benefit from the ability to produce these compounds biosynthetically via large-scale microbial culture, circumventing often expensive and difficult laboratory syntheses. Moreover, understanding the ecological factors that control the production of these metabolites (e.g. physicochemical conditions, cues from potential competitors or grazers) could be used to enhance any such biosynthetic approaches for commercial production.

The possible disadvantage of the application of these compounds as control agents are, likewise, reflected in their ecological relevance. Specifically, as per the previous discussion of quantitative and qualitative defenses, both types of compounds would pose possible limitations to development of such compounds to this end. Quantitative toxins, given their generality of target, would specifically need to be considered in terms of non-specific toxicity that might have implication for both human health (discussed below) and possible ecosystem health. While, on the other hand, qualitative toxins, characterized by very specific targets of action, might lead to populations, and ultimately species, that are able to “co-evolve” adaptations for resistance to these compounds, such that any compound developed would ultimately be prone to subsequent loss of effectiveness in terms of a particular application.

In terms of the potential development and application of any such candidate compound, several considerations will be fundamental. In particular, as mentioned, the specificity of a toxin’s target is perhaps the most important consideration. Any compound that might be characterized by high mammalian toxicity, for example, would have obvious implications for human health, and likely limited potential for widespread commercial use.

Likewise, in addition to possible effects of such compounds in terms of direct human toxicity, potential application of these compounds as environmental control agents would also require consideration of possible implications for ecosystem health. Although compounds with inherent ecological roles in aquatic habitats might be expected to be less disruptive, inappropriate applications could certainly lead to unforeseen consequences as, indeed, would be the case with any environmental control agent. Obviously, such limitations and considerations would differ most significantly between applications in relatively controlled environments (e.g. artificial ponds and pools, agricultural fields) versus natural habitats. In the latter, considerations would specifically include assessment of non-specific activity and environmental persistence as it relates to bioaccumulation (discussed further below), as well as the unique possibility of species displacement in habitats which might have rather grave implications for community structure and consequently ecosystem health. Of course, such considerations are necessary for any such agent, and would need to be considered on a case-by-case basis. However, as discussed previously, development of naturally occurring allelochemicals would benefit from prior and continued ecological assessment of these metabolites.

Finally, considerations for the development of cyanobacterial metabolites as environmental control agents, as with any such candidate compound, is stability and consequent persistence in the environment, as well as the related potential for bioaccumulation. Such a limitation is perhaps best exemplified by the application of the synthetic insecticide, DDT (i.e. dichloro-diphenyl-trichloroethane), used to control mosquito populations after World War II. Though not particularly toxic to mammals, DDT is quite stable – with a biological half-life of approximately eight years – and was found to both persist in the environment, as well as accumulate in the food-web, due to lipophilic nature (and consequent storage in fat tissues). Though relevant data are largely lacking to support any generalization with respect to cyanobacterial metabolites, it might be expected that any such allelochemicals would be short-lived within habitats to avoid any possible effects on subsequent growth of the producing species. Moreover, toxins from cyanobacteria are largely characterized by a diversity of peptide metabolites [4] that might be expected to be relatively labile, particularly at hydrolysable peptide bonds. Given the inability to generalize with respect to stability of cyanobacterial toxins, however, any such efforts to develop these compounds would, of course, require a compound-by-compound assessment of stability and potential bioaccumulation of any such candidate (as would, again, be expected for any candidate compound). Likewise, lipophilicity, given the widely recognized potential for accumulation in fat tissues and subsequent bioaccumulation, might also pose an obstacle for development of such compounds, and (again, as with any such candidate) the uptake and bioavailability (e.g. metabolism, tissue accumulation) of candidate compounds would need to be assessed.

These considerations are perhaps best exemplified by the potential application of allelopathic compounds as algaecides. As mentioned previously, perhaps the greatest obstacle to the effective development of these types of agents is identification of compounds that are taxa-specific, particularly including targets which would make the compounds safe to humans, as well as other “non-target” plants and animals. Allelopathic compounds that specifically target, for example, photosystem II (as discussed above) would seem to fulfill this requirement, however, even this mechanism, specific to photoautotrophs, might still pose a threat to non-pest plants that may be otherwise important to the treated systems, and may even provide an opportunity for resistant taxa, including other potential noxious pest species, to invade and colonize these habitats. Moreover, in many cases, activity such as inhibition of photosynthesis is not the only biological activity, and other modes of toxicity could potentially preclude commercial development as safe algaecides or herbicides. This would be particularly true for any compound with potential for bioaccumulation in the food-web, and ultimate transfer to humans and other higher trophic levels. As an example, the rather lipophilic hapalindoles from *Fischerella* inhibit (as discussed above) photosystem II [103–104], however, further evaluation has shown that these same compounds also generally inhibit RNA synthesis [105], and recent studies [136] have suggested that these compounds may be involved in developmental toxicity observed in zebrafish embryos, as a model of vertebrate toxicity, potentially limiting their safe use as algaecide or herbicide. Similarly, although a number of candidate metabolites that may have potentially allelopathic activity do exist, none have been yet identified which show the specific activity required for development as algaecides or herbicides. Continuing studies of the allelopathic repertoire of cyanobacteria will, however, undoubtedly progress the field toward this end.

Similar to algaecides and herbicides, the deterrence of invertebrate micrograzers by cyanobacterial micrograzers , likewise, poses a potential opportunity for development of commercial products. Though such an approach would be subject to similar considerations (e.g. mammalian toxicity, ecosystem implications, bioaccumulation), as discussed above, the identification and development of cyanobacterial toxins, for control of pest insects, poses a particularly exciting possibility. In particular, controlling mosquito larvae, as a means to control breeding populations of potentially disease-carrying mosquitoes, is emerging as a promising line of research. Taken together, mosquito-borne diseases (e.g. malaria, Yellow Fever, Dengue Fever, various forms of encephalitis, West Nile Virus) kill millions of people worldwide each year, and remain a growing problem [137]. Cyanobacteria represent the largest part of the diet of mosquito larvae [138–139]. In keeping with the aforementioned discussion of chemical defenses of cyanobacteria against micrograzers, it is suggested that production of toxic or deterrent compounds would be selected for by grazing pressures of these larvae, and freshwater cyanobacteria would consequently present a potential source of mosquito larvicidal compounds.

Some limited studies have, in fact, explored this possibility. Kiviranta *et al.* [140] screened extracts from 76 isolates of cyanobacteria and found several of these isolates produced compounds that were larvicidal to *Aedes aegypti*. The greatest inhibition, however, was associated with presence of the hepatotoxic microcystins and the neurotoxic anatoxin-a [140], suggesting limited likely potential for commercial development of practical larvicides from these strains. Subsequently, Nassar *et al.* [141], using *Culex pipiens,* identified larvicidal constituents in crude extracts from cyanobacteria that were active at microgram per larva levels, whereas these concentrations had no apparent mammalian toxicity, specifically measured by serum acetylcholinesterase activity, in a mouse model. Rao *et al.* [142] reported that, while investigating cyanobacteria as a bio-fertilizer, several strains were found to inhibit development of mosquito larvae, and subsequently showed that methanolic extracts from an isolate of *Westiellopsis* sp. were larvicidal to several species of mosquito, including representatives of *Aedes aegypti* (a vector for Dengue Fever), *Anopheles stephensi* (a vector for malaria), and *Culex tritaeniorhyncus* and *C. quinquefaciatus* (vectors of encephalitis), at milligram per liter levels. Perhaps most notably, Harada *et al.* [143] characterized a mixture of unsaturated fatty acids from “*Oscillatoria agardhii* Strain 27” that inhibited larvae of the mosquito species*, Aedes albopictus,* which is closely related to *A. aegypti*. This finding is particularly notable in that the compounds identified included relatively common fatty acids that are not generally considered toxic to mammalian systems.

It should be pointed-out that, in addition to identification and potential development of compounds from cyanobacteria as chemical agents for control of mosquito larvae, an alternative “pro-biotic” approach of disseminating larvicide-producing isolates of cyanobacteria *via* introduction and propagation of these strains in relevant habitats to control populations has also received considerable attention. In fact, though not directly relevant to discussion of cyanobacterial toxins, several studies have explored the use of genetically engineered cyanobacteria, specifically expressing the insecticidal proteins from *Bacillus thuringiensis* to control mosquito larvae [144–150]. Likewise, cyanobacteria that produce naturally occurring larvicidal metabolites could be substituted in this approach, and specifically would eliminate the potential threats associated with release of transgenic organisms. That said, recognizing the potential threat of cyanobacterial toxins to human health, particularly during large-scale blooms, the latter strategy would, of course, present its own concerns. Such an approach would, therefore, require a similar evaluation (as discussed above) of possible mammalian toxicity associated with candidate toxins, as well as ecological implications of the selective introduction into these habitats.

## 6. Allelopathic and Mosquito Larvicidal Compounds from Cyanobacteria in the Florida Everglades

As part of on-going investigation of toxic or otherwise biologically active metabolites from cyanobacteria [104, 136, 151–152], we have isolated and screened over 200 strains of cyanobacteria from the Florida Everglades and other South Florida freshwater sources. In particular, a large majority of these isolates have been specifically isolated from the abundant periphyton mats in the Everglades, representing complex assemblages of microalgae and potential micrograzers, with an equally expected diversity of chemical interactions between the sympatric inhabitants of these complex microcosms. For these studies, cyanobacteria were isolated and cultured, and typically lipophilic (chloroform) and polar (30% ethanol) extracts were prepared, as described previously [136, 151–152]. To date, extracts of these isolates have been screened for cytotoxicity and antimicrobial activity [151–152], as well as toxicity to the zebrafish (*Danio rerio*) embryo as a model system of vertebrate development [136]. This research has enabled identification of novel compounds such as the recently reported pahayokolides [151, 153], as well as possibly new variants of the hapalindole alkaloids [136]. More recently, we have evaluated these isolates for the production of compounds that may have allelopathic activity with respect to sympatric microalgae [104], and report here toxic effects toward mosquito larvae (discussed below).

### 6.1. Antialgal Metabolites from Cyanobacteria and Allelopathy in the Florida Everglades

With respect to the former, seventy-six isolates of cyanobacteria from the Everglades were screened for antialgal activity against two sympatric representatives of green algae (*Selanstrum* 34-4 and *Chlamydomonas* Ev-29) and cyanobacteria (*Anabaena* 66-2 and *Synechococcus* 40-4). Of these, forty isolates (53% of those tested) inhibited one or more of the representative strains. Subsequently, Gantar *et al.* [104] evaluated 8 strains of cyanobacteria, along with 6 strains of green algae, isolated from the Florida Everglades. Specifically, allelopathic activity was assessed, including both inhibition and stimulation of the growth of each “test” strain by each “effector” strain, by means of both co-cultivation studies and evaluation of lipophilic and aqueous extracts from each strain. It was observed that all of the strains evaluated had some degree of inhibition or stimulation with respect to one or more of the paired test strains.

Of these, an isolate (52-1) of the genus, *Fischerella*, had clearly the most pronounced allelopathic activity, and specifically inhibited all of the other 15 strains of microalgae. Moreover, it was found that lipophilic extracts from both biomass and culture medium *Fischerella* 52-1 inhibited photosystem II in the green alga, *Chlamydomonas*, as a model system in a time- and concentration-dependent manner, specifically at concentrations above 1 μg/mL. As discussed above, *Fischerella* has, indeed, been previously documented to produce putatively allelopathic compounds, including the fischerellins and hapalindole alkaloids [103, 105–106] that inhibit photosynthesis and RNA polymerization. In fact, evaluation of lipophilic extracts from *Fischerella* 52-1 culture medium has shown the presence of hapalindole alkaloids [104]. Further supporting (particularly in terms of vertebrate toxicity) the potential for commercial development of these compounds as antialgal agents, inhibition of microalgae [104] occurred at concentrations as low as 100-fold less than those for which toxicity in a vertebrate system (i.e. zebrafish embryos) were observed [136].

### 6.2. Mosquito Larvicidal Activity of Cyanobacterial Isolates

More recently, these cyanobacterial isolates have been screened against mosquito larvae. Both extracts, and lyophilized (i.e. freeze-dried) samples of culture biomass, from cyanobacterial isolates were evaluated for mosquito larvicidal activity, specifically including mortality and abnormal development. For extracts, solutions (in ethanol or 30% ethanol in water for lipophilic and polar extracts, respectively) were added to wells, and solvents evaporated, prior to adding larvae or water; control wells of solvent (i.e. ethanol or 30% ethanol) only were tested along with treatment wells. For evaluation of biomass (i.e. “feeding experiments”), aliquots of approximately 5–10 mg of freeze-dried cells were added to test wells. In these feeding experiments, control groups of mosquito larvae were provided liver powder as the sole food source (as discussed below), whereas “treated” groups of larvae were presented with both cyanobacterial biomass and liver powder. Both experiments were repeated in duplicate (n = 2 x 2 replicates x 4 larvae per replicate).

For assessment of larvicidal activity, mosquitos (*Aedes aegypti*) of the Rockefeller strain were reared at 28°C and 80% relative humidity under a photoperiod cycle of 16 h light/8 h dark. Adult females are offered a cotton wool pad soaked in 3% sucrose solution until 12–16 h before blood feeding. To obtain eggs, three-day-old females are fed pig blood equilibrated to 37°C to which 1 mM ATP is added immediately prior to use. Eggs are collected for 48 h on moist sterile filter paper, and stored in sterile plastic bags at 28°C and 80% relative humidity. For assays, eggs are hatched in double-distilled, deoxygenated water. After approximately fifteen minutes, hatched larvae (four per well) were transferred by pipetting to test wells of 24-well polpropylene plates (Hampton Research, Aliso Viejo, CA) containing distilled water; each larva was transferred with approximately 100 μL of “hatch water” to bring the well volume to 1 mL. Larvae were fed a diet of 3% sterile liver powder added daily (as a 10% suspension), except for Day 2 on which larvae were fed 1.5% liver powder. For feeding experiments, larvae were treated identically to control groups, but additionally presented with 5–10 mg of lyophilized biomass from cyanobacterial isolates. As discussed above, liver powder diet was provided in addition to cyanobacterial biomass to “treated” larvae in feeding experiments, whereas control groups were presented with liver powder diet only.

**Figure 5:**
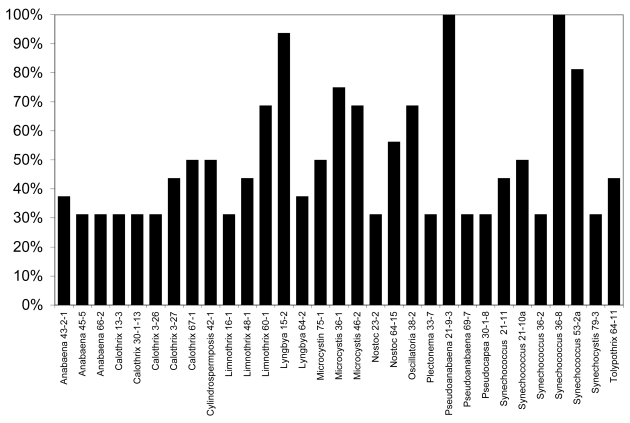
**Strains of Cyanobacteria Isolated from the Florida Everglades and South Florida Resulting in > 25% Mortality of Mosquito (*Aedes aegypti*) Larvae in Feeding Experiments.** Each isolate was tested twice in duplicate (n = 2 x 2 replicates x 4 larvae per replicate), and percent mortality was calculated as average number of dead larvae (divided by four) after 6 days.

None of the lipophilic or polar extracts of cyanobacteria tested were observably lethal or otherwise inhibitory to larval development, however, exposure of larvae to freeze-dried biomass in “feeding experiments” from several of the strains resulted in considerable mortality of larvae (Figure 5). In particular, as shown in Figure 5, 31 of the 120 isolates (25.8%) tested resulted in greater than 25% mortality (i.e. greater than 1 in 4 larvae) after 6 days, whereas no mortality was observed for any of the control larvae (fed liver powder only) in any of the experiments conducted. Of these, nine of the isolates (*Anabaena* 66-2, *Calothrix* 3-27, *Limnothrix* 60-1, *Lyngbya* 15-2, *Nostoc* 23-2, *Plectonema* 33-7, *Pseudoanabaena* 69-7, *Synechococcus* 21-11, *Synechococcus* 21-10a) were previously shown to be toxic to the zebrafish embryo as a model of vertebrate toxicity [136]. Moreover, these conspicuously included a number of isolates from genera with previously described toxicity. Most notably, this included five isolates of *Calothrix* from which apparent RNA polymerase inhibitors, namely the calothrixins, have been previously identified [106–107]. Likewise, isolates of the genera *Anabaena* (3 isolates), *Microcystis* (3 isolates) and *Cylindrospermopsis* (1 isolate) which are producers of the well-described cyanobacterial toxins, anatoxin-a, microcystins and cylindrospermopsin, respectively, showed larvicidal activity, including 50% mortality or higher for larvae exposed to *Microcystis* and *Cylindrospermopsis* isolates.

Of the isolates, the most significant mortality (90–100%) was associated with larvae exposed to biomass of *Lyngbya* 15-2, *Pseudoanabaena* 21-9-3 and *Synechococcus* 36-8. *Lyngbya* 15-2 was previously reported [151] reported to produce a group of cytotoxic peptides, named the pahayokolides, for which the complete structure has been recently published [153]. On the other hand, no compounds have been so far isolated from either *Pseudoanabaena* 21-9-3 or *Synechococcus* 36-8 that both showed 100% mortality of larvae exposed in feeding experiments, and neither isolate showed toxicity toward the zebrafish embryo as a model of vertebrate toxicity [136]. As these studies are based on biomass with undetermined concentrations of active principles, it remains unclear whether the putative allelochemicals are potent or present in high concentrations. Studies are underway to purify and characterize the metabolites.

## 7. Conclusion

Over the past fifty years, a wealth of studies has clarified the importance of secondary metabolites in the ecology of organisms ranging from microorganisms to mammals. Though currently rather limited, growing evidence continues to support a functional role of toxic or otherwise biologically active secondary metabolites from freshwater and marine cyanobacteria in the ecology of these organisms. In particular, these metabolites have seemingly complex roles in the defense against potential grazers and allelopathic interactions with competing photosynthetic microalgae. Still much remains to be elucidated with respect to these roles, and their implications to the evolution of aquatic systems. In addition, however, it has become equally clear that many of these same metabolites may have potential for commercial development of, not only biomedically relevant compounds, but also those with applications for control of algae (and, though not discussed here, potentially other microbes), and control of mosquito larvae and other pests (particularly those that act as potential vectors of disease).

An on-going study of cyanobacteria isolated from the Florida Everglades and other South Florida freshwater sources exemplifies both the suggested role of secondary metabolites in the chemical ecology of these organisms, and the potential for commercial development and application of the bioactive compounds that they produce. A remarkable number of isolates (approximately 53%) were found to inhibit sympatric photoautotrophs, specifically represented by green algae and cyanobacteria, strongly supporting the allelopathic role of cyanobacterial metabolites in this freshwater system. Likewise, a considerable number (approximately 26%) appear to inhibit developing mosquito larvae, possibly through ingestion of cellular material, supporting a role of these compounds in chemical defense of cyanobacteria. Specific isolates have shown particular interest, and are currently being investigated with respect to both chemical ecology and potential commercial development.
